# Human interactome of the influenza B virus NS1 protein

**DOI:** 10.1099/jgv.0.000909

**Published:** 2017-09-04

**Authors:** Corinna Patzina, Catherine H. Botting, Adolfo García-Sastre, Richard E. Randall, Benjamin G. Hale

**Affiliations:** ^1^​ Institute of Medical Virology, University of Zurich, Winterthurerstrasse 190, Zurich 8057, Switzerland; ^2^​ Biomedical Sciences Research Complex, University of St. Andrews, St. Andrews, Fife, KY16 9ST, UK; ^3^​ Icahn School of Medicine at Mount Sinai, 1 Gustave L. Levy Place, New York, NY 10029, USA

**Keywords:** influenza, interferon, virus-host interaction, virulence factor, proteomics

## Abstract

NS1 proteins of influenza A and B viruses share limited sequence homology, yet both are potent manipulators of host cell processes, particularly interferon (IFN) induction. Although many cellular partners are reported for A/NS1, only a few (e.g. PKR and ISG15) have been identified for B/NS1. Here, affinity-purification and mass spectrometry were used to expand the known host interactome of B/NS1. We identified 22 human proteins as new putative targets for B/NS1, validating several, including DHX9, ILF3, YBX1 and HNRNPC. Consistent with two RNA-binding domains in B/NS1, many of the identified factors bind RNA and some interact with B/NS1 in an RNA-dependent manner. Functional characterization of several B/NS1 interactors identified SNRNP200 as a potential positive regulator of host IFN responses, while ILF3 exhibited dual roles in both IFN induction and influenza B virus replication. These data provide a resource for future investigations into the mechanisms underpinning host cell modulation by influenza B virus NS1.

## Abbreviations

AUC, area under the curve; CPE, cytopathic effect; EMCV, encephalomyocarditis virus; FLUAV, influenza A virus; FLUBV, influenza B virus; IFN, interferon; MCS, multiple cloning site; NP40, Nonidet P-40; NS1, non-structural protein 1; PIV5, parainfluenza virus type 5; PKR, dsRNA-activated protein kinase; PI3K, phosphoinositide 3-kinase; SeV, Sendai virus; vRNP, viral ribonucleoprotein complex.

## Full-Text

Although influenza pandemics are historically limited to influenza A viruses (FLUAV) due to their broad host range and consequent potential to acquire antigenically novel surface glycoproteins, seasonal human epidemics are caused by both type A and B influenza viruses. Indeed, influenza B viruses (FLUBV) can be the predominant circulating human influenza virus in some seasons, and have been associated with causing severe disease [1]. Both FLUAV and FLUBV are members of the *Orthomyxoviridae*, and have genomes comprising eight single-stranded, negative-sense RNA segments. The smallest genomic RNA segment of both virus types encodes two proteins, NS1 and NEP/NS2 [2]. The NS1 proteins of both viruses are well described as multi-functional virulence factors that inhibit host interferon (IFN) production and IFN-induced antiviral effectors [3–8]. While the functions and host interactors of the FLUAV NS1 protein have been extensively characterized [9], far less is known about the properties of the FLUBV NS1 protein. Notably, FLUBV NS1, like FLUAV NS1, interacts with dsRNA and inhibits the IFN-inducible dsRNA-activated protein kinase, PKR [4, 10, 11]. However, FLUAV NS1 appears to be unique in targeting specific host-factors such as CPSF30 and the p85β regulatory subunit of PI3K [12–14], while FLUBV NS1 can specifically engage with ISG15 to inhibit antiviral activity [5, 15–17]. In this study, we sought to rationally define the FLUBV NS1 human interactome in order to aid future studies into the molecular mechanisms of influenza B virus–host interactions.

## Characterization of a human cell line stably expressing B/NS1

**Fig. 1.**
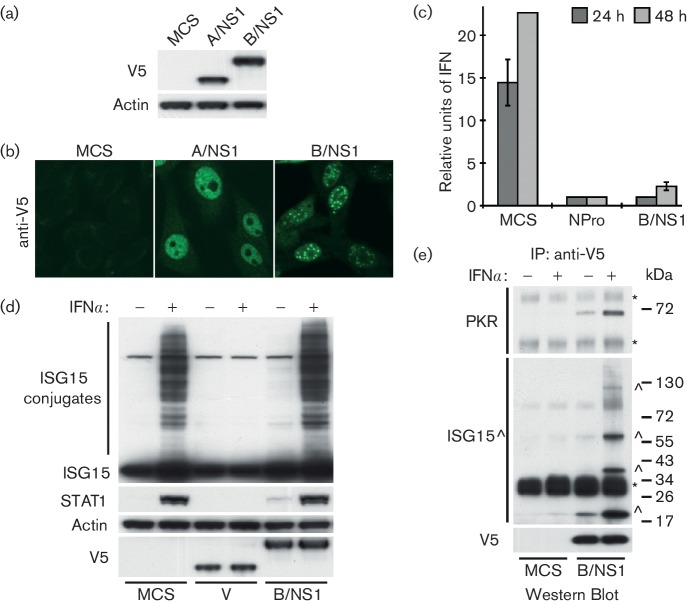
Characterization of a human cell line stably expressing B/NS1. Western blot analysis (a) and immunofluorescence analysis (b) of HEp2 cells stably expressing V5-tagged A/NS1 or B/NS1. (c) B/NS1 blocks IFN induction in response to virus infection. Cells were infected with PIV5(CPI-) at an m.o.i. of 5 p.f.u. cell^−1^, before supernatants were harvested at the indicated times, UV inactivated and titrated by biological EMCV CPE-reduction assay. Mean values of triplicate experiments are shown. Error bars represent standard deviation (SD). (d) Western blot analysis of lysates from HEp2 cells treated, or not, for 20 h with 1000 IU ml^−1^ rIFNα. The indicated proteins were detected with specific antibodies. (e) Specific co-immunoprecipitation of ISG15 and PKR with V5-B/NS1 from lysates of IFN-stimulated (or mock-treated) cells. Asterisks indicate antibody heavy and light chain. ISG15 and potential ISG15-conjugates are indicated by (^). Molecular weight markers (kDa) are indicated to the right.

Using a previously described lentivirus transduction method [14], a puromycin-resistant HEp2 cell line stably expressing an N-terminal V5-tagged form of the FLUBV NS1 protein (strain B/Yamagata/1/73; B/NS1) was generated. In parallel, similar HEp2 cell lines were generated using either an empty multiple cloning site vector (negative control, MCS) or a vector expressing an N-terminal V5-tagged form of the FLUAV NS1 protein [strain A/Puerto Rico/8/34 (PR8); A/NS1]. Compared to parental HEp2 cells, all three cell lines appeared to grow without any obvious deleterious effects. This confirms that B/NS1 is unlikely to alter significantly general host cell gene expression or mRNA processing [10]. The inability of A/NS1 (PR8) to perform these functions is due to a previously documented strain-specific phenotype that many other A/NS1 proteins retain [18, 19]. Western blot analysis confirmed expression of the two V5-tagged viral proteins (Fig. 1a). Immunofluorescence using an anti-V5 antibody directly conjugated to FITC (Bio-Rad, USA) revealed striking differences in the localization of the two NS1 proteins (Fig. 1b). While both proteins localized predominantly (but not exclusively) to the nucleus, A/NS1 was distributed in a largely diffuse pattern but was notably absent in regions likely to be nucleoli [20]. In contrast, B/NS1 primarily localized into discrete punctate intra-nuclear bodies previously characterized as nuclear-speckle domains [21]. During the early stages of FLUBV infection (4–6 h p.i.), the same B/NS1 distribution pattern can be observed. However, at later stages of infection, B/NS1 is found only in the cytoplasm, a re-localization event presumably driven by other viral factors [21]. Thus, the HEp2 cell line stably expressing B/NS1 probably phenocopies the function of this protein in the early post-infection period.

We confirmed that our B/NS1-expressing cells are severely debilitated in their ability to produce IFN (as determined by EMCV CPE-reduction bioassay on Vero cells) in response to infection with the paramyxovirus PIV5 strain CPI- (m.o.i.=5 p.f.u. cell^−1^; Fig. 1c). The block in IFN production is comparable to that observed in HEp2 cells stably expressing the bovine viral diarrhoea virus (BVDV) NPro protein, which targets the essential transcription factor IRF3 for proteasome-mediated degradation [22]. Thus, given that B/NS1 has been reported to prevent the pre-transcriptional nuclear translocation of IRF3 in overexpression experiments [8], these data suggest that either low levels of cytoplasmic B/NS1 are sufficient for this activity, or that B/NS1 (like A/NS1 [18]) has multiple mechanisms for limiting IFN induction, including one possibly occurring in the nucleus. We were unable to observe an effect of B/NS1 on the ability of HEp2 cells to respond to exogenous rIFNα treatment (1000 IU ml^−1^, 20 h), as determined by both formation of ISG15 conjugates and induction of STAT1 protein levels. For this assay, PIV5-V was used as a positive control due to its ability to cause STAT1 degradation [23], thereby blocking IFN signalling and subsequent ISGylation (Fig. 1d). The inability of B/NS1 to block ISGylation was initially surprising given that during overexpression experiments B/NS1 can bind human ISG15 and prevent its conjugation to cellular proteins [15–17, 24]. It may be that the low level of B/NS1 present in the cytoplasm of the HEp2-B/NS1 cells is not sufficient to perform this function or that B/NS1 has a more specific role in counteracting ISG15-mediated antiviral activity, as recently reported by others [5].

## Identification of human proteins interacting with B/NS1

To identify human proteins interacting with B/NS1, including those potentially upregulated by IFN, we took advantage of the observation that HEp2-B/NS1 and HEp2-MCS cell lines responded normally to treatment with exogenous rIFNα, and optimized small-scale immunoprecipitation experiments. Cells were pre-treated for 20 h with 1000 IU ml^−1^ rIFNα in order to upregulate IFN-stimulated genes, before lysing and performing immunoprecipitation of V5-tagged proteins with protein G Sepharose beads coated with anti-V5 antibody as previously described [14] (Fig. 1e). In line with other studies, a robust interaction was observed between B/NS1 and IFN-inducible PKR [25], as well as ISG15 [15, 17]. In addition, it was observed that B/NS1 interacted with specific ISGylated proteins that only appeared after rIFNα stimulation. The identity of these proteins is unknown, but the pattern of interactors is strikingly similar to that observed during FLUBV infection [5]. These data reveal that this system faithfully recapitulates some of the known B/NS1 interactions observed under infection conditions.

**Fig. 2.**
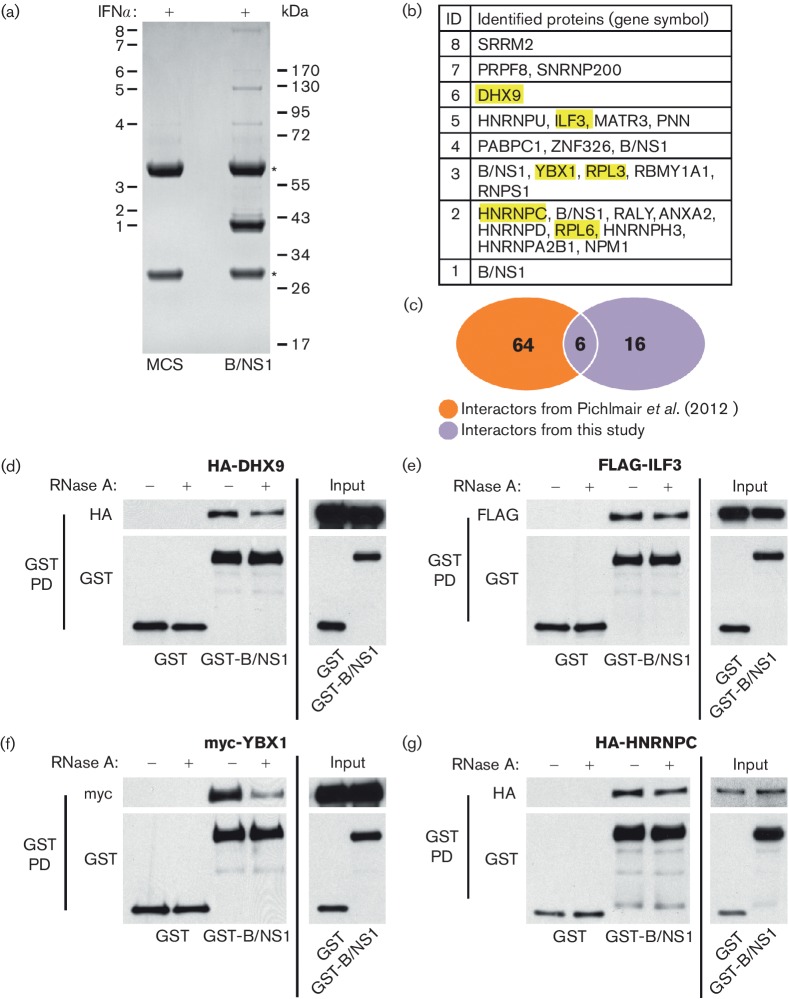
Identification of human proteins interacting with B/NS1. (a) Immunoprecipitation of V5-B/NS1 (or control) from IFN-stimulated cell lysates followed by SDS-PAGE and Coomassie blue staining. Protein bands specific for the B/NS1 lane are numbered. Asterisks indicate antibody heavy and light chain. Molecular weight markers (kDa) are indicated to the right. (b) Proteins identified by mass spectrometry performed on the gel slices cut from A. Each number (1–8) corresponds to the individual band analysed, and gene symbols of the proteins detected are listed. (c) Venn diagram of B/NS1 interactors identified in this study as compared with a previous screen performed by Pichlmair *et al*. The six B/NS1 interactors identified in both screens are highlighted in yellow in (b). (d–g) Western blot analysis of GST-pulldowns from 293 T-cell lysates co-transfected with the indicated plasmids for 48 h. Pull-downs were performed in the presence or absence of RNase A as indicated. Anti-tag antibodies were used to detect proteins of interest.

Large-scale anti-V5 immunoprecipitations were performed from lysates of cells cultured in the presence of rIFNα as previously described [14], except using a modified buffer containing 40 mM Tris-HCl (pH 7.8), 400 mM NaCl, 4 mM EDTA and 0.4 % NP-40. Analysis of the resulting immunoprecipitates by SDS-PAGE and Coomassie blue staining revealed several polypeptide bands that were specific to the B/NS1 pull-down, and were not found in the precipitate from HEp2-MCS cells (Fig. 2a). These polypeptides were excised from the gel and identified by mass spectrometry (bands 1–8). As a negative control for B/NS1 band 4, the corresponding band in the MCS lane was also excised, and the proteins identified in this band by mass spectrometry were excluded from our data set as potential contaminants: namely, HSP70, DDX3, RBM14 and keratin. Overall, 22 B/NS1-interacting human proteins were identified in our study (Fig. 2b and c), including 6 that were previously described out of 70 interactors of the NS1 protein from B/Lee/1940 [26]. Differences in experimental set-up, such as treatment, cell line or virus strain used, most likely explain this low overlap between studies, but nevertheless suggest that the 6 common interactors (DHX9, ILF3, YBX1, RPL3, RPL6 and HNRNPC) are general factors targeted by B/NS1 proteins from different virus strains (see Fig. 2b, highlighted in yellow). Notably, neither this study nor the study of Pichlmair *et al.* identified PKR or ISG15 as B/NS1 interactors by the affinity proteomics approach. The host CrkII adaptor protein, recently identified as a B/NS1 interactor during infection [27], was also not identified by either study. This perhaps indicates sensitivity issues with respect to these proteins using mass spectrometry, or different protein complex stabilities under infection conditions that impact detection capacity.

To validate a subset of these data, we confirmed the specific association of B/NS1 with DHX9, ILF3, YBX1 and HNRNPC by co-transfection of 293 T cells with GST or GST-tagged B/NS1 and the respective tagged host factor constructs [28–30], followed by GST pull-down and identification of co-precipitated proteins by western blot (Fig. 2d–g). These four factors are all known RNA-binding proteins, and therefore by performing the pull-downs either in the absence or presence of RNase A we were also able to show that B/NS1 does not appear to interact indirectly with ILF3 or HNRNPC via common RNA-binding activities (Fig. 2e and g). In contrast, DHX9 and YBX1 exhibited a more striking RNA-dependent interaction with B/NS1 (Fig. 2d and f). These data support the mass spectrometry-based identification of human host factors engaged by B/NS1.

## Functional characterization of components of the B/NS1 interactome

**Fig. 3.**
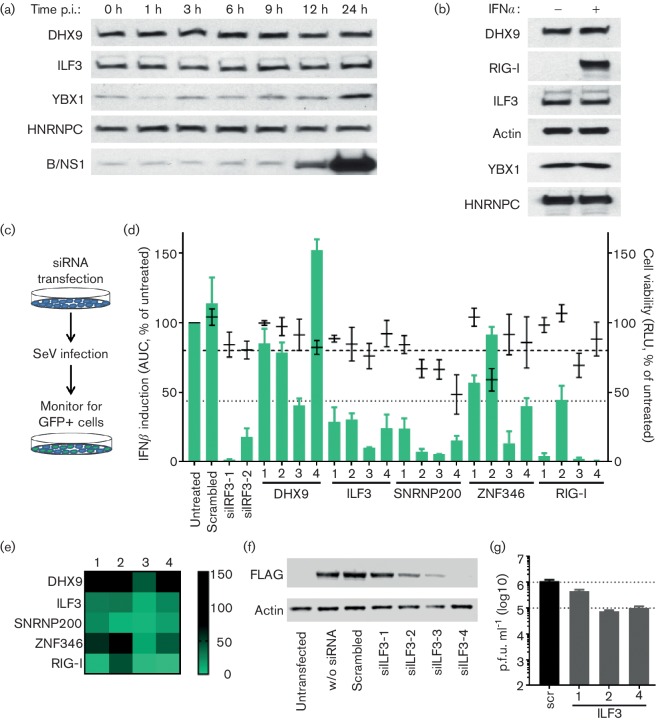
Functional characterization of components of the B/NS1 interactome. (a) Western blot analysis of specific B/NS1 interactors during the course of FLUBV (B/Yamagata/88) infection in A549s (m.o.i. of 5 p.f.u. cell^−1^). (b) Western blot analysis of A549 cell lysates previously treated or not with rIFNα (1000 IU ml^−1^, 20 h). The indicated proteins were detected with specific antibodies. (c–e) Reporter assay for IFNβ-promoter induction. A549-pr(IFNb).GFP cells were transfected for 48 h with specific siRNAs before IFNβ-promoter activation was induced by SeV infection (c). The number of GFP-positive cells was monitored over 24 h, and normalized to overall cell confluency. The area under the curve (AUC) in relation to the untreated control from three independent experiments is depicted (d, green bars, left axis: error bars=sd). Dotted line indicates four sd values away from the negative control. In addition, cell viability after siRNA transfection was measured, and mean relative light units (RLU) from three independent experiments are depicted in relation to the untreated sample (d, black lines, right axis: error bars=sd). Dashed line indicates 80 % cell viability. (e) Heat map representation of the data shown in (d), indicating levels of IFNβ-promoter activation. (f) Western blot analysis of 293 T cells co-transfected (or mock) for 48 h with a plasmid expressing FLAG-ILF3 and the indicated siRNAs. (g) A549 cells were transfected with the indicated siRNAs for 48 h before infection with FLUBV (B/Yamagata/88) at an m.o.i. of 1 p.f.u. cell^−1^. Bars represent mean viral titres in supernatants at 48 h post-infection (*n*=2, error bars represent sd).

Western blot analysis of lysates derived from FLUBV-infected A549s revealed that, from a selected subset of B/NS1 interactors, only YBX1 levels appeared to be upregulated at late times post-infection relative to the other host factors (Fig. 3a). This was not due to YBX1 being IFN-inducible, as none of the selected factors was inducible by rIFNα treatment alone (Fig. 3b). Notably, YBX1, a cellular protein described as being involved in mRNA metabolism [31], has recently been implicated in FLUAV vRNP trafficking and budding [32, 33]. A similar involvement of YBX1 in the FLUBV replication cycle remains to be determined, as does the function of YBX1 protein upregulation and its interplay with B/NS1 during the course of infection.

B/NS1 is well described in regard to counteracting IRF3 signalling and the induction of IFN, potentially at the level of the RIG-I sensor, and possibly via two independent mechanisms involving B/NS1 domains that can both bind RNA [4, 8, 34, 35]. Given that neither this study nor Pichlmair *et al.* identified classical components of the RIG-I/TRIM25/MAVS signalling axis as B/NS1 interactors, we investigated whether another B/NS1 interactor impacted IFN induction, and could therefore be a target for B/NS1 antagonism. In this regard, an siRNA screen was performed to determine the contribution of selected B/NS1 interactors to Sendai virus (SeV)-induced activation of the IFNβ-promoter. B/NS1 interactors were selected with known RNA-binding or helicase activities from the proteomic screens: DHX9 and ILF3 as hits from both screens, SNRNP200 as a unique hit from our screen and ZNF346 as a unique hit from the screen of Pichlmair *et al*. For the assay, a previously established A549-based reporter cell line that expresses GFP under control of the IFNβ-promoter [36] was used. Cells in 96-well plates were independently transfected with four individual siRNAs per factor (or positive/negative controls, all from Qiagen) for 48 h prior to stimulation of the IFNβ-promoter with SeV. Cells were then continuously monitored for green fluorescence over a 24 h period using the IncuCyte Live-Cell Analysis System (Essen BioScience) (Fig. 3c). The number of GFP-expressing cells was automatically counted and normalized to overall cell density. The relative total area under the curve (AUC) for each condition was then taken as an indirect measure of IFNβ-promoter activation, with untreated, SeV-infected cells set to 100 % induction. This level of induction would represent an approximate 2000-fold increase in *IFNβ* mRNA over baseline if quantified by qPCR (data not shown). The screen was performed three times independently, and parallel, uninfected plates were used to determine potential siRNA toxicity as measured by cell viability (CellTiter-Glo Luminescent Cell Viability Assay, Promega). To define a ‘hit’ that impacted IFNβ-promoter activation, a stringent threshold was chosen of four standard deviations away from the untreated, SeV-infected condition (considering all samples from all independent experiments) that had to be observed for at least three out of four individual siRNAs (Fig. 3d and e). Using these criteria, RIG-I and IRF3 were confirmed to play essential roles in SeV-induced activation of the IFNβ-promoter, while no significant impact could be determined for DHX9 or ZNF346. However, all four siRNAs targeting the B/NS1 interactors SNRNP200 and ILF3 showed a strong impact on IFNβ-promoter induction, suggesting important roles for these factors in IFN production (Fig. 3d and e).

Another group recently reported that SNRNP200 contributes to the IFN induction pathway by a mechanism involving its interaction with viral RNA and the cellular adapter protein, TBK1, leading to activation of IRF3 signalling [37]. However, ILF3 (also known as NF90) has not been previously implicated directly in regulating IFN induction, although it has been reported to have some antiviral activity against FLUAV [38–40]. We therefore investigated whether ILF3 also influences the replication of FLUBV. It was confirmed that all four siRNAs targeting ILF3 were able to reduce ILF3 levels in co-transfected cells (Fig. 3f), and that three out of four siRNAs did not reduce cell viability >20 % (Fig. 3d). Next, A549 cells were transfected with the three non-toxic siRNAs for 48 h before infecting them with FLUBV (strain B/Yamagata/88) at an m.o.i. of 1 p.f.u. cell^−1^ . At 48 h post-infection, supernatants were collected and virus levels were titrated via standard plaque assay. As shown in Fig. 3(g), under these conditions, targeting ILF3 with 3 independent siRNAs negatively affected FLUBV propagation up to 1-log, with impact on virus replication correlating, to a certain extent, with the knockdown capacity of each siRNA. These data suggest that ILF3, despite its potential role in IFN induction pathways, plays an important role in FLUBV replication. Such apparently discordant observations have been noted previously, as ILF3 and ILF3-related proteins can exhibit context-dependent pro- or anti- viral activities [41–46]. Thus, the precise contribution of ILF3 to the IFN response in FLUBV-infected cells, as well as its interplay with both B/NS1 and general viral replication, require further investigation.

## Concluding Remarks

Herein, we expanded the known human host interactome of the isolated FLUBV NS1 protein, a multifunctional virulence factor heavily understudied in comparison to FLUAV NS1. It will be critical to confirm these interactions in the context of virus infection, and to determine their relative importance. Nevertheless, this functionally validated dataset is a valuable new resource to complement future studies into the mechanism of action of how this viral protein manipulates host-cell processes, as well as its potential roles in directly influencing virus replication and counteracting host innate immune responses.
